# Near-infrared fluorescence imaging for sentinel lymph node identification in colon cancer: a prospective single-center study and systematic review with meta-analysis

**DOI:** 10.1007/s10151-019-02107-6

**Published:** 2019-11-18

**Authors:** M. Ankersmit, H. J. Bonjer, G. Hannink, L. J. Schoonmade, M. H. G. M. van der Pas, W. J. H. J. Meijerink

**Affiliations:** 1 Department of Surgery, Cancer Center Amsterdam, Amsterdam UMC-Vrije Universiteit Amsterdam, De Boelelaan 1117, Amsterdam, The Netherlands; 2 Department of Operating Rooms and MITeC Technology Center, Radboud University Medical Center, Nijmegen, The Netherlands; 3 Medical Library, Amsterdam UMC, Vrije Universiteit Amsterdam, Amsterdam, The Netherlands; 4 Department of Surgery, Meander Medisch Centrum, Amersfoort, The Netherlands

**Keywords:** Sentinel lymph node, Indocyanine green, IRDye800CW, Colon cancer

## Abstract

**Background:**

Near-infrared (NIR) fluorescence imaging has the potential to overcome the current drawbacks of sentinel lymph node mapping (SLNM) in colon cancer. Our aim was to provide an overview of current SLNM performance and of factors influencing successful sentinel lymph node (SLN) identification using NIR fluorescence imaging in colon cancer.

**Methods:**

A systematic review and meta-analysis was conducted to identify currently used methods and results. Additionally, we performed a single-center study using indocyanine green (ICG) as SLNM dye in colon cancer patients scheduled for a laparoscopic colectomy. SLNs were analyzed with conventional hematoxylin-and-eosin staining and additionally with serial sectioning and immunohistochemistry (extended histopathological assessment). A true-positive procedure was defined as a tumor-positive SLN either by conventional hematoxylin-and-eosin staining or by extended histopathological assessment, independently of regional lymph node status. SLN procedures were determined to be true negatives if SLNs and regional lymph nodes revealed no metastases after conventional and advanced histopathology. SLN procedures yielding tumor-negative SLNs in combination with tumor-positive regional lymph nodes were classified as false negatives. Sensitivity, negative predictive value and detection rate were calculated.

**Results:**

This systematic review and meta-analysis included 8 studies describing 227 SLN procedures. A pooled sensitivity of 0.63 (95% CI 0.51–0.74), negative predictive value 0.81 (95% CI 0.73–0.86) and detection rate of 0.94 (95% CI 0.85–0.97) were found. Upstaging as a result of extended histopathological assessment was 0.15 (95% CI 0.07–0.25). In our single-center study, we included 30 patients. Five false-negative SLNs were identified, resulting in a sensitivity of 44% and negative predictive value of 80%, with a detection rate of 89.7%. Eight patients had lymph node metastases, in three cases detected after extended pathological assessment, resulting in an upstaging of 13% (3 of 23 patients with negative nodes by conventional hematoxylin and eosin staining).

**Conclusions:**

Several anatomical and technical difficulties make SLNM with NIR fluorescence imaging in colon cancer particularly challenging when compared to other types of cancer. As a consequence, reports of SLNM accuracy vary widely. Future studies should try to standardize the SLNM procedure and focus on early-stage colon tumors, validation of tracer composition, injection mode and improvement of real-time optical guidance.

**Electronic supplementary material:**

The online version of this article (10.1007/s10151-019-02107-6) contains supplementary material, which is available to authorized users.

## Introduction

Colon cancer is one of the most common malignancies in the Western world and the number of early-stage tumors (T1 and T2) identified is expected to increase as a result of the introduction of nationwide screening programs [1]. Lymph node metastases are the strongest predictive factor for patient survival [2]. The low risk of lymph node metastasis in these early-stage tumors makes local excision of the primary tumor an attractive treatment option [3]. However, current treatment by segmental resection with en bloc resection of lymph nodes is unavoidable as long as uncertainties about undetected lymph node metastasis remain. Despite complete segmental resection, up to 20–30% patients with early-stage disease will develop distant metastasis and eventually die from colon cancer [4]. This high recurrence rate in node-negative colon tumors could be the result of understaging due to missed occult tumor cells (e.g., isolated tumor cells or micrometastases) during routine histopathological examination or inadequate lymph node harvesting [5–8]. Detailed examination of all lymph nodes using serial sectioning and immunohistochemistry (IHC) is desirable. However, these techniques are time consuming and expensive and are, therefore, not appropriate for daily practice. On the other hand, the majority of colon cancer patients without lymph node metastasis are exposed to unnecessary surgery-related morbidity and mortality, currently of 13.5% and 2.0%, respectively [9].

The concept of sentinel lymph node mapping (SLNM) in colon cancer as a staging technique has been described frequently, with variable results [10]. The limited penetration depth and fast migration of current blue dyes, resulting in high false-negative rates, are frequently mentioned as serious drawbacks of SLNM. Near-infrared (NIR) fluorescence imaging for SLNM has several properties that are advantageous for the SLN procedure in colon cancer and has already shown promising results in several types of cancer [11–13]. The relatively high penetration depth and real-time optical guidance are important benefits in SLN identification since lymph node drainage patterns of colon cancer are unknown and SLNs are generally in an unfavorable location beneath a fatty mesocolon.

Since this technique has increased interest in SLNM in colon cancer, it is important to obtain a broader understanding of the technique and of the factors that influence the success of NIR fluorescence imaging for SLN identification. Therefore, a systematic review and meta-analysis was conducted to provide an overview of the current performance of SLNM with NIR fluorescence imaging in colon cancer, and of the factors influencing its sensitivity, detection rate, negative predictive value and upstaging rates. These outcomes are compared with results and experience of a prospective single-center study performed in our hospital which aimed to determine SLNM accuracy using NIR fluorescence imaging in 30 colon cancer patients.

## Materials and methods

### Systematic literature review and meta-analysis

**Fig. 1:**
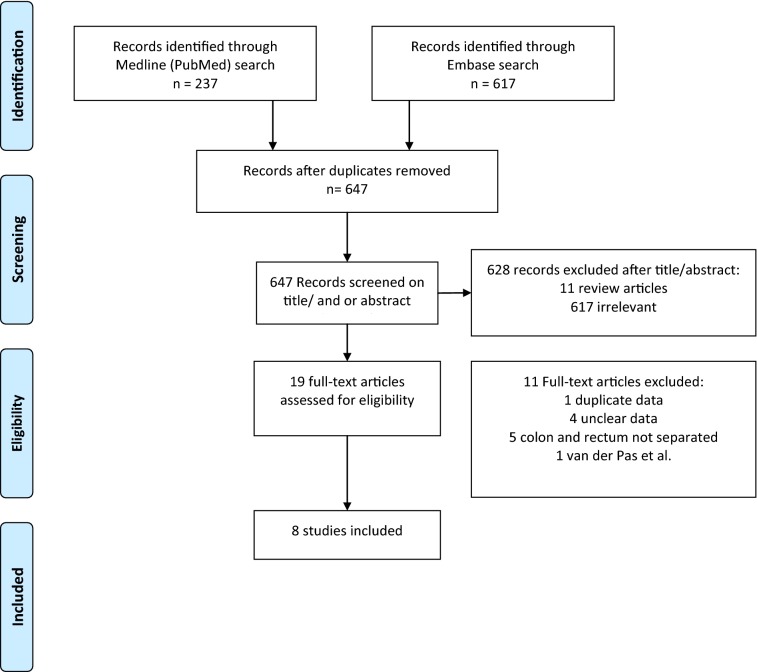
Flow diagram of study selection

The protocol for the review was registered in the international prospective register of systematic reviews (PROSPERO, registration number CRD42018110076). A systematic literature search was performed in PubMed MEDLINE and Embase from inception through October 2nd, 2018, in collaboration with a medical librarian (LS). The literature search was in compliance with the screening guidelines of the Preferred Reporting Items for Systematic Reviews and Meta-Analysis (PRISMA) (Fig. 1) [14]. Two authors (MA and WJHJM) independently conducted the literature search. In case of disagreement regarding inclusion or exclusion, a paper was discussed to establish consensus. The following medical subject headings (MeSH) were used as index terms: ‘colorectal neoplasms’, ‘Sentinel Lymph Node Biopsy’, ‘Indocyanine Green’ and ‘Fluorescent dyes’. The full PubMed search strategy is detailed in Supplementary Information 1. Systematic reviews and narrative review articles identified during the search were checked for additional references. Articles were included if they fulfilled the following criteria: (1) description of the use of NIR imaging for SLNM in colon cancer; (2) prospective design to assess the effectiveness of identification and diagnostic performance of the SLN procedure in colon cancer; (3) separate results for colon and rectal cancer. Studies that performed SLNM only in rectal carcinoma were excluded. To avoid overlapping patient data in duplicate publications, the article with the largest sample size was included. Studies published in English, German or Dutch were included. Articles were systematically screened by title, followed by abstract screening and finally full-text screening.

Variables collected consisted of gender, age, body mass index (BMI), number of patients with colon cancer, T stage of disease, tumor size, in vivo or ex vivo injection, tracer injection site, tracer composition, tracer concentration, injected dose, investigator’s definition of SLN, total number of SLNs, total number of regional lymph nodes, number of SLN procedures, number of failed SLN procedures, histopathological technique(s) used, imaging system, and any adverse effects of SLNM. The quantitative results were used to build 3 × 2 contingency tables to estimate numbers of true positives (TP), true negatives (TN) and false negatives (FN) (Supplementary Information 2, Fig. 1). A true positive procedure was defined as a tumor-positive SLN with or without advanced histopathology analysis, independently of regional lymph node status. In this context, a false-positive rate was zero by definition since false-positive histopathological findings are not possible. SLN procedures were determined to be true negatives if SLNs and regional lymph nodes revealed no metastases after conventional and advanced histopathology. SLN procedures yielding tumor-negative SLNs in combination with tumor-positive regional lymph nodes were classified as false negatives.

As false positives were not possible, the specificity and positive predictive value of the SLN procedure were 100% by definition. The diagnostic parameters sensitivity, negative predictive value, detection rate, and upstaging were recalculated from the included data.

Sensitivity of the SLN procedure was defined as the number of true positives in patients with positive histopathological findings (TP/TP + FN). The negative predictive value was defined as the number of patients in whom a negative SLN correctly predicted the lymph node status of the total lymph node yield (TN/TN + FN). Detection rate was the proportion of successful SLN procedures divided by all executed SLN procedures. Patients were considered as ‘upstaged’ in case of positive SLNs at advanced histopathology without tumour-positive regional lymph nodes with conventional histopathology. The percentage of upstaged patients was calculated by dividing the number of upstaged patient with the number of TN patients after conventional histopathology (upstaged patients/upstaged patients + TN).

Quality assessment of the studies was based on the revised tool for the quality assessment of diagnostic accuracy studies (QUADAS-2) [15]. Risk of bias was independently assessed by two reviewers (MA and WJHJM). Discrepancies in interpretation were resolved by discussion (Table 1).

**Table 1:**
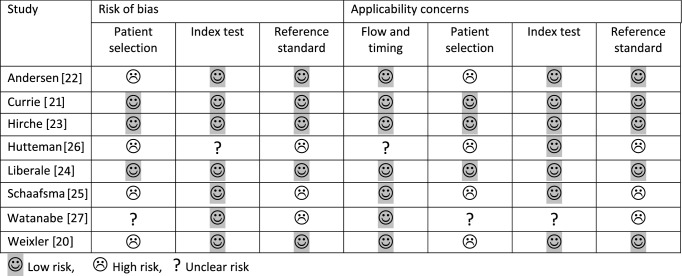
Results of quality assessment of the studies included according to QUADAS-2

We used the data from the 3 × 2 tables to calculate sensitivity, negative predictive value, detection rate and upstaging. Individual results were presented graphically by plotting these values with their 95% confidence intervals in forest plots. A bivariate random-effects approach for the meta-analysis of sensitivity [16] and negative predictive value was used [17]. We investigated heterogeneity visually by examining the forest plots and statistically by including covariates in the bivariate models through conducting subgroup analysis. The following sources of heterogeneity were assessed: (1) used tracer (indocyanine green, IRDye800CW whether or not combined with blue dye); (2) number of injections (2–4 injections vs. random number of injections); (3) injection technique (in vivo or ex vivo); (4) injection site (subserosal vs. submucosal); (5) time between injection and SLN mapping (directly after injection, 3–10 min after injection and > 15 min after injection). We incorporated these factors as covariates in the bivariate models to examine the effect of potential sources of bias and variation across subgroups of studies.

Pooled estimates of detection rates and upstaging with their corresponding 95% confidence intervals were calculated using Freeman–Tukey double arcsine transformation within a random effects model framework. Heterogeneity of combined study results was assessed by *I*^2^, and its connected Chi-square test for heterogeneity, and the corresponding 95% confidence intervals were calculated. Statistical analysis was performed using R version 3.6.0 (R Foundation for Statistical Computing, Vienna, Austria) with packages “mada” and “meta.”

### Prospective single-center study

We performed a prospective single-center study which was approved by the Medical Ethics Committee of the UMC-Amsterdam, Vrije Universiteit Amsterdam, The Netherlands. The study was registered in the Clinical trials database (NCT02122523). Included patients were at least 18 years old with proven colon cancer and scheduled for laparoscopic colectomy. All patients provided oral and written informed consent. Exclusion criteria included pre- and peroperative gross lymph node invasion, distant metastases, prior colorectal surgery, advanced disease with invasion of adjacent structures, metastatic or T4 disease on preoperative imaging or discovered during intraoperative staging, rectal cancer, contraindications for laparoscopy and allergy to iodine. The first 14 patients received a transcutaneously performed subserosal injection of ICG and the subsequent 15 patients had submucosal tracer injection by colonoscopy.

Injection of ICG was performed in vivo in all patients after general anesthesia. The injection solution consisted of 25 mg ICG diluted in 1.0 ml human albumin (20%) and 9.0 ml NaCl (0.9) [18]. Injection of dye followed prior injection of 1.0 ml NaCl (0.9%) to ensure correct needle placement in the submucosal layer in both injection techniques. A subserosal injection consisted of 1–3 peritumoral injections with a sclerosing needle. For submucosal injection, a colonoscopy was executed directly after general anesthesia and placement of a laparoscopic port. ICG was administrated by 1–4 submucosal injections at the base of the tumor using a V960 injection needle (Prince Medical, Gutenberg, France). In both groups, the surgical procedure started with the exposure of the peritoneal cavity and operative field, followed by mobilization of the colon medial to lateral. The mesocolon was inspected with conventional imaging and additionally with a NIR 30° laparoscope (Olympus, Tokyo, Japan). All fluorescent lymph nodes identified with the NIR camera were considered SLN(s), intraoperatively harvested and presented to the pathologist separately from the rest of the specimen. All lymph nodes were bisected along the longest axis, paraffin embedded and stained with H&E. Serial sectioning (3–4 µm thick) at 150-µm intervals was followed by H&E staining. Immunohistochemistry for the epithelial carcinoembryonic-antigen (CEA) (Clone 1117; DAKO Netherlands M7072), CAM 5.2 (3,455,799; BD Biosciences Netherlands) and CK19 (M0888, clone RCK 108; DAKO The Netherlands) followed when no metastases were found after routine H&E staining. Metastases were classified according to the TNM 5 guidelines. Metastases identified by serial sectioning and IHC were classified as reported by Hermanek and colleagues [19]. Sensitivity, detection rate, negative predictive value and upstaging were calculated by comparing the results of SLNM and pathological examination for all lymph nodes and expressed as a median (range).

## Results

### Systematic review and meta-analysis

We identified eight eligible studies published between January 2006 and August 24th, 2018, presenting 227 SLN procedures [20–27]. Since no additional articles were found by cross-checking references, these eight studies were analyzed and critically appraised. The selection process is shown in Fig. 1. We excluded the study of van der Pas et al. [28] because these results were reported in our single-center study. An overview of included articles is given in Tables 2 and 3. None of the studies reported adverse events.

**Table 2:** Characteristics of included studies

Study	Patients	Successful procedures	Male:female	Age (years)	BMI (kg/m^2^)	Tumor diameter (mm)	T1	T2	T3	T4	Tumor location	Number LNs	Number SLNs
Andersen [22]	29	19	18:11	69 (36–79)	26 (19–31)	na	1	7	19	2	Cecum to sigmoid	24 (9–24)	1 (0–3)
Currie [21]	30	27	na	69 (61–73)	26.2 (19–31)	37 (29–49)	6	8	14	2	Cecum to sigmoid	34 (27–39)	3 (1–4)
Hirche [23]	26	25	na	67 (47–87)	28.4	na	6	5	14	1	Cecum to sigmoid	32.9 (10–143)	1.7 (0–5)
Hutteman [26]	19	19	na	64 ± 16.6	27.5 ± 6.17	42 ± 13	1	4	11	3	Cecum to sigmoid	16.2 ± 5.34	3.2 ± 1.01
Liberale [24]	20	20	9:11	70 (43–87)	26.3 (18–36)	39 (0–100)	5	1	10	4	Cecum to sigmoid	22.4 (5–41)	1.5 (0–4)
Schaafsma [25]	22	21	12:10	69 (41–88)	25 (20–40)	37 (9–90)	2	7	10	3	Cecum to sigmoid	20.5 ± 8.1	3.5 ± 1.9
Watanabe [27]	31	31	22:9	67.5 ± 12.2	23.6 ± 3.24	na	13	18	0	0	Splenic flexure	17.5 ± 7.6	10.4 ± 4.73
Weixler [20]	50	49	31: 19	68 ± 11.2	na	na	7	7	35	1	Cecum to sigmoid	23.4 ± 9.5	4.4 ± 2.2

*BMI* body mass index, *LNs* lymph nodes, *SLNs* sentinel lymph nodes, *na* not available

**Table 3:** Technical characteristics of SLN mapping of included studies

Study	Infrared system	Fluorescent tracer	Tracer composition	Concentration	Injected dose	Injection technique	Site of injection	Time of SLN mapping	Histopathological examination for SLNs
Andersen [22]	SPIES (Karl Storz, Holte, Denmark)	ICG; ICG Pulsion, Pulsion Medical Systems, Munich, Germany	ICG: HAS diluted in H2O and blue dye	ICG 25 mg H2O 9 ml HSA 1 ml	2 × 0.5 ml proximal an distal from the tumor	In vivo	Subserosal	Intraoperative directly and 20 min after injection	Five series of 50 um interval, 3 um thickness/each; 1st section H&E and 3rd section with IHC for cytokeratin A
Currie [21]	Laparoscopic NIR-imaging system (Olympus Corporation, Tokyo, Japan)	ICG; ICG Pulsion, Pulsion Medical Systems, Munich, Germany	na	5 mg/ml	4 × 1 ml circumferentially	In vivo	Submucosal	Intraoperative 7 (IQR 6–8) min after injection	Standard H&E-staining. If negative for metastases, serial sectioning in slices of 4 um at 250 um intervals, staining of each level with H&E and IHC for pan-cytokeratin antibody
Hirche [23]	IC-View (ICG Pulsion, Pulsion Medical Systems, Munich, Germany)	ICG stock solution	na	5 mg/ml	2.0 (range 1–4) ml around the tumor	In vivo	Subserosal	Intraoperative 3–10 min after injection and ex vivo	H&E staining at 250 um. If negative for metastases, re-examination by serial sectioning at 5 um and H&E staining and IHC for cytokeratin antibody for each section
Hutteman [26]	Mini-FLARE	IRDye800CW; Li-Cor, Licoln, NE	IRDye800CW: HSA diluted in PBS	3:1	1 ml circumferentially	Ex vivo	Submucosal	Ex vivo 5 min after injection and tracer massage	H&E staining at 4-um sections
Liberale [24]	Photodynamic Eye PDE (Hamamatsu, Japam)	ICG (Pulsion, Paris, France)	ICG diluted in H2O and blue dye	0.5 mg/ml	4 × 0.5 ml circumferentially	Ex vivo	Subserosal	Ex vivo directly after injection and during pathological examination	Standard H&E staining. If negative for metastases, serial sectioning using three slices at 150 um interval stained with H&E, if still negative IHC for anti-pan-cytokeratin
Schaafsma [25]	Mini-FLARE	IRDye800CW; Li-Cor, Lincoln, NE	IRDye800CW: HSA diluted in PBS and blue dye	1.5:1	1 ml circumferentially	Ex vivo	Submucosal	Ex vivo 5 min after injection and tracer massage	H&E staining at 4-um sections
Watanabe [27]	In vivo: D-Light P System (Karl Storz, Tuttlingen, Germany) Ex vivo: HyperEye Medical System, Mizuho corporation, Tokyo, Japan	Diagnogreen; Daiichi Pharmaceuticals, Tokyo, Japan	ICG diluted in H2O	2.5 mg/ml	2 × 1 ml proximal and distal from the tumor	In vivo	Subserosal	Intraoperative 30 min after injection and ex vivo	H&E staining
Weixler [20]	Mini-FLARE	IRDye800CW; Li-Cor, Lincoln, NE	IRDye800CW: HSA diluted in PBS and blue dye	3:1	0.4 ± 0.2 ml Number of injections depends on tumor size	Ex vivo	Subserosal	15 min after injection	Serial sectioning at 3 levels, H&E at the 1st section of each level. If negative for metastases then IHC for cytokeratin 19 for a second section

*SLN* sentinel lymph node, *na* not available, *H&E* hematoxylin and eosin, *IHC* immunohistochemistry

BMI was reported in seven studies and varied widely from 19 to 40 kg/m^2^ [21–27]. None of the studies mentioned BMI as a potential factor of influence on SLN performance. Four studies described tumor size, which varied between 9 and 100 mm [21, 24–26]. All studies disclosed tumor stage. Early-staged T1 and T2 tumors were found in 41 (18%) and 57 (25%) patients, respectively. T3 tumors were diagnosed in 113 (50%) reported patients and T4 tumors in 16 patients (7%).

As fluorescent mapping agent, ICG was used in five studies sourced from different companies [21–24, 27]. ICG was dissolved in distilled water in three studies [22, 24, 27] and humanized-serum albumin (HSA) was added in one study [22]. Three studies used IRDy800CW conjugated to HSA and dissolved in PBS [20, 25, 26] (Li-Cor, Lincoln, NE,USA). In four studies, injection of a fluorescent tracer was combined with the administration of blue dye [20, 22, 24, 25]. The concentration of the fluorescent dyes varied between 0.5 and 5.0 mg/ml. In all studies, injection occurred around the tumor. The number of injections varied between 2 and 4 injections proximal and distal to the tumor [22, 23, 27] or circumferentially [21, 24], up to a random number of injections depending on tumor size [20, 25, 26]. A lower volume of tracer was injected when the number of injections was determined by tumor size compared to those using two–four standard injections. Administration of tracer occurred in vivo [21–23, 27] or ex vivo [20, 24–26]. Procedures using IRDye800CW as a tracer were all performed ex vivo since the dye was not Food and Drug Administration approved during the performance of the studies. Both techniques allow subserosal or submucosal injection. The submucosal injection technique was used in three studies [21, 25, 26] and subserosal in five studies [20, 22, 23, 24, 27]. SLNs were identified directly after dye injection [22, 24], after 3–10 min [21, 23, 25, 26] or more than 15 min after injection [20, 27]. As shown in Table 2, the highest number of SLNs was identified when SLNM was performed more than 15 min after injection.

In five studies, SLNs were serial sectioned and stained with IHC when no (sentinel) lymph node metastasis was found after conventional H&E staining [20–24]. Intervals used in the serial sectioning of 3–5-um sections varied between studies and ranged from 50 to 250 um. A cytokeratin antibody was used for IHC in all five studies, as cytokeratin is highly expressed in metastases of colorectal origin.

**Fig. 2:**
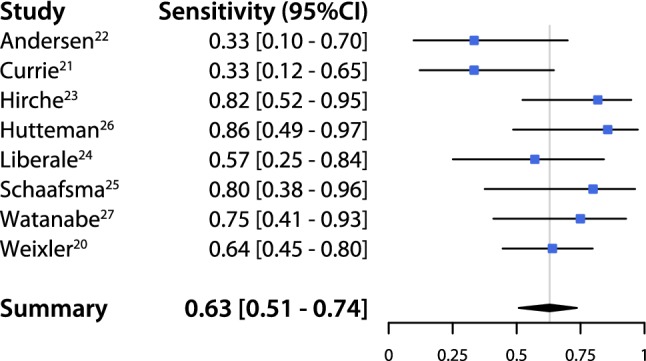
Pooled sensitivity

**Fig. 3:**
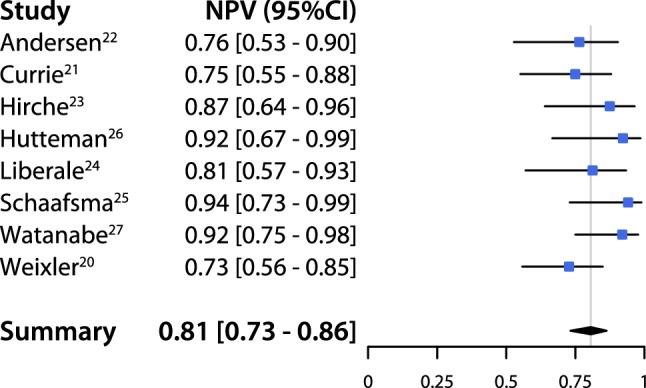
Pooled negative predicitive value

**Fig. 4:**
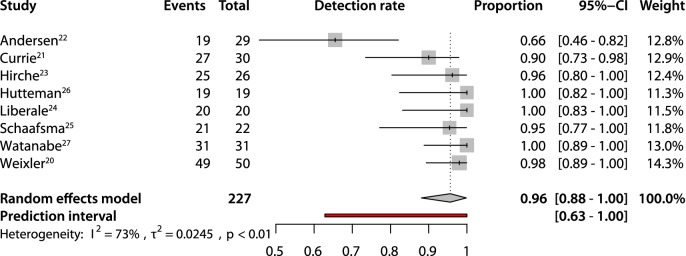
Pooled detection rate

**Fig. 5:**
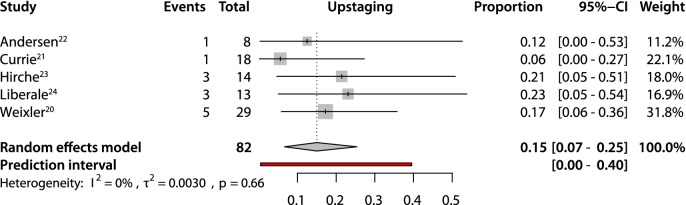
Pooled upstaging

An overall pooled sensitivity of 0.63 (95% CI 0.51–0.74) and negative predictive value of 0.81 (95% CI 0.73–0.86) were estimated (Figs. 2, 3). The pooled detection rate was estimated to be 0.96 (95% CI 0.88–1.0) (Fig. 4). Upstaging could be calculated in five studies [20–24] and a pooled upstaging of 0.15 (95% CI 0.07–0.25) was estimated (Fig. 5). Thus, 15% of patients with negative SLNs assessed with conventional histopathology showed occult tumor cells (isolated tumor cells or micrometastases) after examination with advanced histopathology. None of the subgroup analyses showed statistically significant differences between subgroups in terms of sensitivity, negative predictive value or detection rate (Supplementary Information, Table 2).

### Prospective single-center study

A total of 30 patients were included. One patient was excluded due to intraoperatively detected gross lymph node metastasis. Patient characteristics and results for each patient are shown in Table 4. No adverse events related to ICG were noticed. Subserosal dye injection occurred in 14 patients and submucosal injection in 15 patients. In two patients (patients 22 and 28), no fluorescent SLNs were intraoperatively identified resulting in an overall detection rate of 89.7%, with a median of 2 (range 0–6) SLNs and 13 (range 1–34) regional lymph nodes identified. Of the 29 patients, nine patients showed lymph node metastases. Five of these patients showed metastases in the regional lymph nodes but not in the SLN (patients 1, 2, 4, 10, 17). These SLNs are considered as false negatives. In one patient, lymph node metastases were found in both the SLNs and regional lymph nodes (patient 27). These SLNs are classified as true positives. In three procedures, SLNs showed metastases in only the SLNs which were found at advanced histopathology (patients 15, 16 and 18). In two of these patients, SLNs contained isolated tumor cells found with additional IHC (patients 16 and 18) and in one patient SLN metastases (> 2 mm) were detected after serial sectioning and H&E staining (patient 15). These three patients were converted from node negatives to node positives and, therefore, considered as ‘upstaged’ and also true positives.

**Table 4:** Individual patient results

Patient	Diagnosis	Tumour location	BMI (kg/m^2^)	Tumour size (cm)	Injection side	Number of injections	SLN found	Malignant SLN	Non-SLNs	Malignant regional lymph nodes	SLN status
1	T3N1M0	Ascendens	25.9	7.0	SS	1	3	0	34	1	FN
2	T3N2M0	Cecum	25.9	7.0	SS	1	1	0	22	8	FN
3	T2N0M0	Transversum	24.5	2.5	SS	1	2	0	1	0	TN
4	T3N1M0	Sigmoid	22.9	10	SS	2	2	0	6	1	FN
5	T3N0M0	Sigmoid	22.2	4.5	SS	1	4	0	5	0	TN
6	T2N0M0	Cecum	31.2	4.0	SS	1	2	0	9	0	TN
7	T2N0M0	Cecum	30.1	3.0	SS	1	2	0	25	0	TN
8	T3N0M0	Sigmoid	26.4	9.5	SS	2	1	0	4	0	TN
9	T3N0M0	Sigmoid	25.8	4.5	SS	1	3	0	10	0	TN
10	T3N1M0	Sigmoid	21.0	3.5	SS	3	2	0	4	2	FN
11	T3N0M0	Ascendens	25.1	0.8	SS	1	1	0	27	0	TN
12	T1N0M0	Cecum	22.0	6.0	SS	1	6	0	32	0	TN
13	T2N0M0	Cecum	23.8	4.5	SS	3	2	0	14	0	TN
14	T3N0M0	Sigmoid	25.1	3.5	SS	3	6	0	14	0	TN
15	T3N1M0	Sigmoid	25.6	4.5	SM	4	4	1	12	0	TP/Upstaging
16	T3N0M0	Transversum	23.3	5.5	SM	2	2	2	21	0	TP/Upstaging
17	T3N1M0	Sigmoid	26.1	6.5	SM	4	4	0	17	3	FN
18	T2N0M0	Sigmoid	26.3	4.0	SM	1	4	1	11	0	TP/Upstaging
19	T1N0M0	Transversum	29.3	0.9	SM	1	0	0	30	0	TN
20	T3N0M0	Sigmoid	29.0	5.0	SM	4	3	0	15	0	TN
21	T2N0M0	Sigmoid	22.9	0.8	SM	1	4	0	8	0	TN
22	T3N0M0	Sigmoid	29.0	5.0	SM	3	0	0	12	0	TN
23	T1N0M0	Cecum	35.2	4.5	SM	1	2	0	13	0	TN
24	T1N0M0	Sigmoid	27.1	2.0	SM	1	2	0	13	0	TN
25	T1N0M0	Cecum	24.4	2.2	SM	1	2	0	14	0	TN
26	T1N0M0	Sigmoid	29.3	2.0	SM	1	2	0	7	0	TN
27	T4N1M0	Transversum	43.1	7.0	SM	4	4	2	29	1	TP
28	T1N0M0	Transversum	33.0	2.3	SM	1	0	0	5	0	TN
29	T1N0M0	Sigmoid	27.9	3.3	SM	3	3	0	9	0	TN

*BMI* body mass index, *SLN* sentinel lymph node

As a result, the sensitivity of the procedure was 44% (4/9), negative predictive value 80% (20/25) and upstaging 13% (3 of 23 patients with negative nodes by conventional hematoxylin and eosin staining).

Results for sensitivities after subserosal injection and submucosal injection were 0 versus 80%, respectively. Negative predictive values were 71% after subserosal injection and 91% using the submucosal injection technique.

## Discussion

The concept of SLNM to improve lymph node staging in colon cancer has been investigated extensively [10]. Due to the wide variation in reported outcomes and frequent lower accuracy of results compared to breast cancer and melanoma, overall treatment decision-making based on SLN assessment is still not safe. The location of colonic SLNs in the fatty mesocolon and limited penetration depth of blue dye through fatty adipose tissue are frequently mentioned as serious obstacles to accurate SLNM. NIR imaging using a fluorophore as mapping agent has been proposed as a more effective technique for SLNM in colonic malignancies due to its high tissue penetration of up to 1 cm [29].

In this meta-analysis, we included eight studies that described the use of a fluorophore as a mapping agent for SLNM in colon cancer. We found wide variation between studies regarding the NIR fluorescent SLNM techniques employed, resulting in large differences in reported outcomes. Subgroup analyses were, therefore, performed to identify the technically related factors influencing SLNM accuracy. These technical factors included the tracer used, injection site, in vivo or ex vivo SLNM performance, number of injections and timing between tracer administration and SLN identification. Additionally, tracer composition, dosage and concentrations all varied between studies. Pooled detection rates and negative predictive values both showed acceptable results, at 0.96 (95% CI 0.88–1.0) and 0.81 (95% CI 0.73–0.86), respectively. However, an overall low sensitivity of 0.63 (95% CI 0.51–0.74), accompanied by wide variation (33–85%), was reported among included studies. Although subgroup analysis showed comparable results for SLN accuracy, we believe that several technical factors contributed to the poorer results for SLNM using NIR fluorescence imaging in colon cancer compared to those reported in other types of cancer [30].

The first technical factor which should be validated is site of injection for tracer administration. Although meta-analysis results showed no significant differences after subserosal or submucosal injection of dye, notably outcomes in terms of sensitivity and negative predictive value were found after subserosal injection in our single-center study. According to the current literature, this can be explained by the greater accuracy of injection near the tumor after submucosal injection, which also probably improves uptake by all tumor-draining lymphatic vessels [26, 31–33]. Another possible explanation is difficulty regarding correct needle positioning and maintaining position during tracer administration. We have personally experienced this problem, especially when using the subserosal injection technique. To improve the accuracy of needle positioning, an injection of 1.0 ml saline in the colon wall was administrated before ICG. The raised `bleb` was helpful in confirming correct positioning of the needle into the colon wall. However, an unfavourable side effect of the injection of saline prior to ICG is the increase of hydrostatic pressure at the injection site. This resulted in dislocation of the needle in several patients and extravasation of dye into the peritoneum, which then made SLN identification impossible in these patients due the high intrinsic background fluorescence in the abdominal cavity. We experienced no spillage of dye using the submucosal injection technique. Only one of the reviewed studies described spillage of dye, which in that case occurred after injection into the subserosal layer, but the authors did not mention this as a cause of false-negative SLN procedures [22]. Independently of the injection technique used, correct needle placement and careful administration of tracer without spillage of dye is crucial to a successful SLN procedure. There appears to be a steep learning curve for accurate tracer administration and we, therefore, recommend that this procedure should only be performed by an experienced surgeon or gastroenterologist.

Tracer administration and SLN identification can be performed in vivo or ex vivo. Advantages of the ex vivo SLN procedure include avoidance of patient exposure to dye, interruption of the surgical procedure, and the relative simplicity of the technique. In addition, ex vivo SLNM is thought to allow a more aggressive dissection of the mesocolon, resulting in improvement of SLN identification [34]. However, ex vivo SLN identification after extraction of the specimen disrupts natural lymphatic pathways. Moreover, an ex vivo approach may exclude SLNs located outside the resection area, whereas an in vivo SLN procedure might have identified aberrant lymph node drainage patterns and thus changed the mesocolonic resection margins [35]. In our study, we did not modify our resection margins, but several reviewed studies identified metastatic SLNs which would not have been included in the standard resection specimen [22, 27, 36]. Furthermore, an in vivo approach can potentially facilitate SLN picking, combined with local excision of the primary tumor when no metastases are found in the SLNs. This treatment approach would dramatically change therapy options for patients with early-staged tumors, and could potentially decrease surgery-related morbidity rates while improving patient survival [3]. It must be emphasized that node picking interrupts the standard oncological approach. Second, a higher number of lymph nodes retrieved are associated with prolonged disease-free survival due to better lymph node staging [37]. These facts underline the need for a highly sensitive SLNM technique before minimization of surgery can be justified in cases where no metastases are found in the SLNs [38].

Optimal timing of tracer administration is another technical factor which needs to be improved. Despite the helpful larger diameter of ICG or possibility of using HSA, the tracer still shows fast migration to higher echelon lymph nodes, resulting in a greater number of fluorescent regional lymph nodes. Designating a large number of fluorescent lymph nodes as SLNs is undesirable since advanced histopathological examination must be applied to all these nodes, which is expensive, time consuming and probably not cost-effective. Another contributing factor is time of harvesting between injection and SLN detection. As shown by the results in Table 2 of the reviewed studies, numbers of assigned SLNs were highest when SLNM was performed more than 15 min after injection [20, 27]. A high number of SLNs should be avoided, as non-SLNs could be mistaken for SLNs and unnecessary costs will be incurred. Therefore, surgeons should attempt to follow lymph flow drainage patterns directly after injection which would overcome prolonged time intervals between tracer administration and SLNM.

A wide variety of dye compositions, concentrations and dosages were employed in the reviewed studies. It is important to note that ICG and IRDye800CW should be seen as distinct tracers, as they have their own specific chemical and physical properties. An ICG dose of around 500 ug has been suggested as optimal for SLNM but this has never been clearly investigated in colon cancer [12, 39–41]. Humanized-serum albumin is frequently added to ICG and IRDye800CW, which may improve metabolic activity and result in a brighter fluorescent signal. In addition, HSA should increase the hydrodynamic diameter, leading to better retention of dye in the SLN. However, results for fluorescent tracer combined with HSA in SLNM are inconclusive [18, 42, 43, ]. More research to optimize concentration, dosage and composition is necessary to improve the fluorescent signal and to allow differences in signal intensity between the SLN and surrounding tissue to be distinguished.

To improve the NIR fluorescence imaging SLN procedure in colon cancer, patient and tumor- related characteristics should be investigated in addition to technical factors. Currie et al. [21] reported a sensitivity of 33% and argued that this was caused by inclusion of large tumors (> 35 mm) and high number of T3 and T4 tumors, which are associated with more advanced tumor stages. Weixler et al. [20] and Liberale et al. [24] agreed that the inclusion of T3–T4 tumors is a potential cause of high false-negative rates, but not tumor size itself. Tumor stage and size are both frequently mentioned as causes of high false-negative rates in the current literature [44]. It is well-established that higher stage disease with more advanced transmural tumors increases the risk of lymph node metastasis. Second, large longitudinal and transserosal tumors could theoretically destroy efferent lymphatic pathways and may involve adjacent lymphatic drainage patterns, which can increase false-negative rates. It must be emphasized that these factors have never been verified as confounders in large studies [10, 45]. However, since more advanced tumor stages already meet criteria for adjuvant chemotherapy, and treatment of these tumors will not be altered by a SLN procedure, we suggest that future studies should try to include only early-stage tumors.

High BMI and additional mesocolonic adiposity are associated with a decreased sensitivity for SLNM [45, 46]. Although none of the included studies confirmed this association, we experienced technical difficulties during SLNM in patients with a fatty mesocolon. First, it must be emphasized that effective penetration of fluorescence is still limited in fatty mesocolon. Additionally, more fatty tissue requires more dissection, leading to additional risk of disrupting the lymphatic vessels, with leakage of dye into the abdominal cavity as a consequence. As a result, SLN identification becomes more challenging since adhesion of dye to fat compromises the NIR view and fluorescing fat could be mistaken for an SLN [21].

Finally, the pathological assessment of SLNs has a major influence on the accuracy of the procedure. Only advanced histopathological techniques are able to detect occult tumor cells (e.g., isolated tumor cells and micrometastases), although the prognostic significance of these occult tumor cells is still unclear. Nevertheless, it has been suggested that micrometastases are associated with a significant reduction in 5-year survival [5, 8]. Therefore, studies of the SLN procedure should use serial sectioning and IHC for histopathological examination to all SLNs when no lymph node metastasis is found after conventional H&E staining [47].

Limitations of the results presented here include the marked variability in the methods used and the small number of patients included in each study, including our own single-center study.

## Conclusions

Evidence regarding SLNM with NIR fluorescence imaging in colon cancer is still limited. Better standardization of the technique will be necessary in future trials. These studies should concentrate on early-stage tumors and focus on tracer composition, injection mode and the improvement of real-time optical guidance to the SLN. Moreover, due to several anatomical and technical difficulties, the SLN procedure in colon cancer seems to be more challenging compared to other types of cancer, and considerable expertise will be required before large patient-related studies can be undertaken to validate SLNM as part of the standard surgical treatment in colon cancer.

## Electronic supplementary material

Below is the link to the electronic supplementary material.

Supplementary material 1 (DOCX 14 kb)

## Data Availability

The datasets during and/or analyzed during the current study are available from the corresponding author on reasonable request. All data generated or analyzed during this study are included in this published article and its supplementary information files.
