# Supplementary material for: Near-infrared fluorescence imaging for sentinel lymph node identification in colon cancer: a prospective single-center study and systematic review with meta-analysis
Source: Tech Coloproctol. 2019 Nov 18;23(12):1113–26. doi: 10.1007/s10151-019-02107-6 (PMC6890578; doi:10.1007/s10151-019-02107-6)
Supplement: Supplementary file 1 — Supplementary material 1 (DOCX 14 kb) [file 10151_2019_2107_MOESM1_ESM.docx]

**SUPPLEMENTARY INFORMATION 2**

Table 1. Contingency table 3 x 2

|  | LNs with metastases after H&E staging | LN without metastases |
| --- | --- | --- |
| SLNs with metastases after H&E staging | True positives (TP) | True positives (TP) |
| SLNs without metastases after H&E staging | False negatives (FN) | True negatives (TN) |
| SLNs with metastases after serial sectioning and immunohistochemistry | True positives (TP) | True positives (TP)/ upstaged patients |

Table 2. Outcomes of tracer composition and injection technique to primary outcomes

| Variable | Studies | Sensitivity 95%CI | Negative predictive value 95%CI | Detection rate  95%CI |
| --- | --- | --- | --- | --- |
| Tracer |  |  |  |  |
| Fluorescent tracer alone | 4 | 0.69 (0.41-0.88) | 0.85 (0.73-0.83) | 0.98 (0.91-1.0) |
| ICG | 5 | 0.57 (0.36-0.76) | 0.81 (0.72-0.88) | 0.94 (0.80-1.0) |
| IRDye800CW | 3 | 0.69 (0.52-0.82) | 0.86 (0.63-0.95) | 0.98 (0.93-1.0) |
| Additional blue dye | 4 | 0.60 (0.45-0.74) | 0.78 (0.70-0.86) | 0.93 (0.76-1.0) |
| Number of injections |  |  |  |  |
| 2-4 injections | 5 | 0.57 (0.56-0.76) | 0.81 (0.72-0.88) | 0.94 (0.80-1.0) |
| Random | 3 | 0.69 (0.52-0.82) | 0.86 (0.63-0.95) | 0.98 (0.93-1.0) |
| Injection technique |  |  |  |  |
| In vivo | 4 | 0.57 (0.30-0.81) | 0.81 (0.71-0.89) | 0.91 (0.74-1.0) |
| Ex vivo | 4 | 0.67 (0.52-0.80) | 0.82 (0.68-0.91) | 0.99 (0.95-1.0) |
| Injection site |  |  |  |  |
| Subserosal | 5 | 0.64 (0.51-0.76) | 0.80 (0.71-0.87) | 0.95 (0.83-1.0) |
| Submucosal | 3 | 0.66 (0.27-0.91) | 0.86 (0.67-0.95) | 0.96 (0.87-1.0) |
| SLN mapping |  |  |  |  |
| Directly after injection | 2 | 0.47 (0.22-0.73) | 0.79 (0.62-0.90) | 0.88 (0.41-1.0) |
| 3-10 min after injection | 4 | 0.67 (0.54-0.78) | 0.84 (0.73-0.91) | 0.96 (0.89-1.0) |
| More than 15 min after injection | 2 | 0.66 (0.49-0.80) | 0.83 (0.54-0.95) | 0.99 (0.95-1.0) |
